# Noisy-channel language comprehension in aphasia: A Bayesian mixture modeling approach

**DOI:** 10.3758/s13423-025-02639-z

**Published:** 2025-01-28

**Authors:** Rachel Ryskin, Edward Gibson, Swathi Kiran

**Affiliations:** 1 University of California, Merced, 5200 N Lake Rd., Merced, CA 95343 USA; 2 Health Sciences Research Institute at UC Merced, Merced, USA; 3 Massachusetts Institute of Technology, Cambridge, USA; 4 Boston University, Boston, USA

**Keywords:** Aphasia, Language comprehension, Noisy-channel

## Abstract

Individuals with “agrammatic” receptive aphasia have long been known to rely on semantic plausibility rather than syntactic cues when interpreting sentences. In contrast to early interpretations of this pattern as indicative of a deficit in syntactic knowledge, a recent proposal views agrammatic comprehension as a case of “noisy-channel” language processing with an increased expectation of noise in the input relative to healthy adults. Here, we investigate the nature of the noise model in aphasia and whether it is adapted to the statistics of the environment. We first replicate findings that a) healthy adults (*N* = 40) make inferences about the intended meaning of a sentence by weighing the prior probability of an intended sentence against the likelihood of a noise corruption and b) their estimate of the probability of noise increases when there are more errors in the input (manipulated via exposure sentences). We then extend prior findings that adults with chronic post-stroke aphasia (*N* = 28) and healthy age-matched adults (*N* = 19) similarly engage in noisy-channel inference during comprehension. We use a hierarchical latent mixture modeling approach to account for the fact that rates of guessing are likely to differ between healthy controls and individuals with aphasia and capture individual differences in the tendency to make inferences. We show that individuals with aphasia are more likely than healthy controls to draw noisy-channel inferences when interpreting semantically implausible sentences, even when group differences in the tendency to guess are accounted for. While healthy adults rapidly adapt their inference rates to an increase in noise in their input, whether individuals with aphasia do the same remains equivocal. Further investigation of comprehension through a noisy-channel lens holds promise for a parsimonious understanding of language processing in aphasia and may suggest potential avenues for treatment.

Extracting meaning from a sentence often seems so easy as to feel automatic for healthy listeners and readers. Yet, this fluency belies the complex inferences which take place with every instance of sentence comprehension. For example, English readers rely on the semantic context of a sentence or larger discourse to infer the intended meaning of an ambiguous word (e.g., Rodd, Gaskell, & Marslen-Wilson, 2002). Similarly, readers use the position of words and other syntactic cues (e.g., the presence of function words, morphology, etc.) to understand the roles and relations that a sentence describes. For instance, “The dog chased the fox” can be understood to mean that the dog was doing the chasing and the fox was being chased even though the reverse is also semantically plausible given world knowledge. In most cases, the semantic and syntactic cues agree and provide partially redundant information (Mahowald, Diachek, Gibson, Fedorenko, & Futrell, 2022). But, in some sentences, the semantic information is insufficient or even conflicts with the structural information (e.g., The bunny chased the fox). These sorts of sentences pose a unique challenge for individuals with aphasia (IWA) (Caramazza & Zurif, 1976; Schwartz, Saffran, & Marin, 1980), in particular those termed ‘agrammatic.’ The speech of individuals with agrammatism is characterized by the production of grammatically ill-formed utterances. In comprehension, when faced with conflicting semantic and syntactic cues, individuals with agrammatism appear to rely more heavily on plausibility than healthy individuals – they are more likely to say that the fox chased the bunny.

The standard explanation for IWA’s over-reliance on plausibility during sentence comprehension held that this was a manifestation of a deficit in core syntactic knowledge that was functionally tied to the location of the patient’s lesion, typically in Broca’s area (e.g., Caramazza & Zurif, 1976; Grodzinsky, 1990). However, several sources of evidence have since cast doubt on this view. For instance, the link between lesion location and functional deficit appears to be more complex (Dick et al., 2001) than originally proposed (Caramazza, 1986). Further, IWA often retain sensitivity to grammatical violations when they are asked to explicitly identify them (e.g., Linebarger, Schwartz, & Saffran, 1983; Wulfeck & Bates, 1991), and which sentences/structures pose the greatest challenge often varies depending on the type of task (Caplan et al., 2006), suggesting that syntactic knowledge itself may be largely spared (for discussion see Saffran & Schwartz, 1994). Further, healthy adults under cognitive or processing load can behave like IWA with agrammatism (Blackwell & Bates, 1995; Kilborn, 1991; Miyake, Carpenter, & Just, 1994), suggesting that aphasic language processing may be on a continuum with healthy adult language processing, rather than representing a sharp break from it (cf. Badecker & Caramazza, 1985).

Alternative accounts of agrammatic comprehension propose that deficits in memory or computational resources cause IWA to fail to parse a complex sentence (for overview, see Papagno & Cecchetto, 2019). Sentences with complex syntactic structures (e.g., long-distance dependencies) are more difficult to process even for healthy readers, as evidenced by slow downs in reading (e.g., Gibson, 1998; Grodner & Gibson, 2005). Individual differences studies suggested that the sentence processing of individuals with lower working memory capacity was more disrupted by additional structural complexity (Just & Carpenter, 1992; King & Just, 1991) compared to that of individuals with higher working memory capacity. Additionally, working memory was thought to support the re-analysis of a sentence when the initial parse leads to a semantically incoherent interpretation (e.g., Caplan, Michaud, & Hufford, 2013). By extension, it was proposed that individuals with agrammatic aphasia may struggle to process the structural elements of sentences not because of impairment to a syntactic module, but because their working memory capacities were diminished relative to their healthy counterparts (Miyake et al., 1994). However, several studies fail to find support for the relationship between individual differences in working memory and the ability to process syntactically complex sentences (e.g., Caplan, DeDe, Waters, Michaud, & Tripodis, 2011; James, Fraundorf, Lee, & Watson, 2018; Traxler, Williams, Blozis, & Morris, 2005). Others have argued that domain-general working memory capacity deficits do not accurately describe the neurocognitive profiles of individuals with aphasia and propose alternative cognitive resources which may play a role in sentence processing, such as distinct phonological and semantic working memory stores (Martin, 1995) and cognitive control (Novick, Kan, Trueswell, & Thompson-Schill, 2009). In sum, whether agrammatic comprehension is caused by limitations in non-linguistic cognitive resources remains debated.

In part, the lack of consensus may be related to the coarse classifications (e.g., agrammatic, anomic) often used to categorize IWA and the implied discontinuity between these classifications as well as between all IWA and healthy adults (for discussion see e.g., Badecker & Caramazza, 1985; Ralph, Patterson, & Plaut, 2011; Schwartz & Dell, 2010). Detailed multimodal evaluations of individuals classified as agrammatic suggest that there is substantial variability within this classification. The same behavior – the lack of reliance on syntax during sentence comprehension – may have distinct causes across individuals (e.g., deficits in cognitive control for some and over-reliance on semantic information for others, Thothathiri, Kirkwood, Patra, Krason, & Middleton, 2023).

Consistent with a more nuanced view of agrammatic comprehension, Gibson et al. (2013) observed that, when semantic and syntactic cues conflict (e.g., in an implausible sentence like “The mother gave the candle the daughter”), healthy readers often interpret sentences according to a related, more plausible meaning (the candle being given to the daughter), rather than according to the syntactically correct literal meaning. They proposed that – far from being evidence of a deficit – this reflects a rational process of inferring meaning from sentences transmitted through ‘noise,’ and that the same rational inference process might explain the comprehension patterns of IWA (Gibson et al., 2016). By providing a computational-level framework that takes into account semantic, syntactic, and noise information, and allows for individual differences in how that information is integrated, noisy-channel has the potential to offer a unifying account for healthy and aphasic comprehension, which may open new avenues for investigating the neural mechanisms underlying aphasia (e.g., the role of cognitive control).

## The noisy-channel framework for language comprehension

Following (Shannon, 1948), the noisy-channel framework views language comprehension as a process of rational Bayesian inference given uncertain input (Gibson et al., 2013; Levy, 2008; Levy, Bicknell, Slattery, & Rayner, 2009). All human communication is subject to noise, whether its because of a speech error, background conversations at a café, or the waxing and waning of attention, among other possibilities. This means that a speaker or writer’s intended sentence $S_i$ may be corrupted during transmission and differ from the sentence, $S_p$, which is perceived by the comprehender. Rational comprehenders implicitly account for this possibility by inferring $S_i$ through Bayesian reasoning, as in (1)

1 $$\begin{aligned} P(S_i | S_p) \propto P(S_p|S_i) \cdot P(S_i) \end{aligned}$$

The left-hand side of Eq. (1), $P(S_i|S_p)$, is the posterior probability assigned by the comprehender to an intended sentence $S_i$ given what they perceived, $S_p$. According to Bayes’ rule, this is proportional to the product of the prior probability $P(S_i)$ that the producer intended to communicate $S_i$ and the likelihood, $P(S_p|S_i)$, that $S_p$ would be perceived given that $S_i$ was intended. In other words, when inferring the meaning of a received sentence, the comprehender weighs how probable the sentence and all its alternatives are given language and world knowledge against the likelihood of the noise corruptions that could have transformed $S_i$ into $S_p$ during communication.

Gibson et al. (2013) demonstrated that this noisy-channel explanation is consistent with human sentence comprehension behaviors in healthy adults. Participants read semantically implausible but syntactically licit sentences, such as “The mother gave the candle the daughter,” and answered comprehension questions (e.g., Did the daughter receive something?). A large proportion of readers answered the comprehension question based on the non-literal meaning of the sentence (i.e., choosing “yes”). This behavior is consistent with readers inferring that the target sentence might have resulted from the deletion of “to” from the more plausible sentence, “The mother gave the candle to the daughter,” and answering the question relative to this alternative. Such an inference would be probable based on Eq. (1), because $P(S_p|S_i)$, the probability of a deletion of “to” via a typographical error or misreading, is likely relatively high, and the prior $P(S_i)$ of this alternative intended sentence would be much higher than $P(S_i)$ of the target sentence.^1^

Further, the rate of these noisy-channel inferences differed by sentence type: sentences where the noise that would have to be posited was less likely, in terms of Levenshtein distance^2^ between the perceived sentence and the closest alternative, led to fewer non-literal inferences. For example, the exchange of “girl” and “ball,” which would motivate interpreting “The girl was kicked by the ball” according to a more plausible meaning, is a less likely corruption and implausible sentences like these elicited fewer inferences than those which only required assuming the deletion of a preposition like “to.”

Similar to neurotypical language users, IWA also appear to engage in noisy-channel processing during comprehension (Gibson et al., 2016; Warren, Dickey, & Liburd, 2017). When they hear sentences that are semantically implausible but syntactically correct, they tend to infer that the more semantically plausible alternative was intended (originally characterized as the use of a “heuristic” interpretation strategy, Caramazza & Zurif, 1976), and they do so more readily when the noise corruption has higher likelihood. The critical difference is that IWA make more inferences overall, suggesting that they assign a higher likelihood to any noise operations relative to healthy controls. Understanding why this might be promises to shed light on key properties of the syndrome of aphasia, as well as human language processing in general, and suggest new avenues for intervention which target the comprehender’s estimate of the noise likelihood.

## Present work

In the present research, we investigate noisy-channel inference during sentence processing in healthy adults and IWA with a focus on individual differences in both populations. In particular, we use a hierarchical Bayesian modeling approach, which allows us to capture variability in the proclivity for noisy-channel inference across individuals and provides a more fine-grained view of sentence processing in healthy adults and IWA with and without agrammatism.

We also examine how the noise likelihood may differ in IWA relative to healthy controls leading them to engage in more frequent noisy-channel inference. In healthy adults, the estimates of noise likelihood are malleable. When readers find themselves in an environment where there are many typographical errors, they increase their rate of noisy-channel inferences (Gibson et al., 2013). Similarly, when the speaker has a foreign accent, listeners increase their reliance on semantic plausibility (Gibson et al., 2017), suggesting that top-down, contextual information plays a role in adapting the noise likelihood. This adaptation can be more fine-grained than a simple increase or decrease: readers track the distribution of the kinds of noise (e.g., deletions, insertions, exchanges) in their environment (Ryskin, Futrell, Kiran, & Gibson, 2018) and interpret sentences accordingly.

We hypothesize that the IWA’s noise likelihood differs from that of healthy adults as a result of adapting to the functional consequences of their neurological damage. The nature of this adaptation is an open question. For instance, IWA may have a higher base-rate of noise in their noise model because of perceptual (hearing, vision) difficulties (i.e., they assume that they are often mis-hearing/mis-reading), or because they themselves produce errors in speaking (and their own errors form part of their input), or because the people speaking to them tend to modify their speech in particular ways intended to make it easier to comprehend (commonly known as “elderspeak” and often involving simplifying syntax, Kemper, Ferrell, Harden, Finter-Urczyk, & Billington, 1998).

We go beyond the previous work in multiple ways. First, the sample sizes in previous work with IWA are relatively small (*N* = 8 in Gibson et al., 2016 and *N* = 16 in Warren et al., 2017) and, as a result, many of the effect sizes may be over- or under-estimates (e.g., most of the reported models used sub-optimal random effects structures in order to reach convergence). Here we use a larger sample size and, importantly, an analysis approach that allows us to use the model that we think best describes the generative process of the data without being hampered by convergence issues, provides information about the amount of uncertainty around the estimated effect sizes, can capture multiple latent effects as well as individual differences, and precludes the need to exclude participants based on arbitrary criteria such as accuracy on filler trials (see Overall Analysis Approach below). This analytical approach provides a novel perspective on noisy-channel comprehension with healthy adults as well as IWA. Previous work with healthy readers (e.g., Gibson et al., 2013) has not jointly modeled the effect of different edit types and noise, nor the role of individual differences, despite the observation that participants in these experiments vary in terms of whether they mostly endorse literal interpretations or mostly endorse the inferred interpretations. The prior work also typically excludes inattentive participants based on an arbitrary cut-off related to filler accuracy, which only affects a small number of healthy participants in online studies but is likely to be substantially more problematic for IWA.

Second, the conclusions drawn in the previous work with IWA are limited by the fact that the tasks used in Gibson et al. (2016) and Warren et al. (2017) may have created memory demands that are not present in Gibson et al. (2013) by using auditory presentation. Previous accounts of comprehension deficits in aphasia have posited a key role for memory resources (Caplan et al., 2013). While noisy-channel inference likely operates over memory representations as well (e.g., Futrell, Gibson, & Levy, 2020; Hahn, Futrell, Levy, & Gibson, 2022), in order to understand the basic ways in which individuals with aphasia arrive at the meaning of a sentence and isolate factors that contribute to the noisy-channel explanation, it is useful to minimize all other cognitive resource demands. In the present studies, participants read sentences and answer comprehension questions, presented on the same display, entirely at their own pace. They are able to re-read each sentence as much as they want.

Third, we investigate whether IWA adapt their noise model to the rate of noise in the local context. If IWA are able to rapidly adapt their noise likelihood to the properties of the input, this would suggest a potential avenue for treatment, by calibrating their noise model more appropriately to the noise rates that exist in everyday communication settings through exposure. However, the increased noise rate may reflect a more global, top-down adaptation to the individuals’ circumstances that is insensitive to the statistics of the input. Consequently, we don’t have a strong expectation that a short exposure in the lab will change how IWA interpret sentences. Nonetheless, this manipulation will contribute to increasing the generalizability of our findings by testing whether rates of noisy-channel inference in IWA hold across different linguistic contexts (one with errors and one without).

In two experiments, IWA and healthy controls (including an age-matched sample) participated in a reading comprehension task modeled on Gibson et al. (2013). The critical sentences were syntactically well-formed but semantically implausible (e.g., “The mother gave the candle the daughter”), and responses to comprehension questions (e.g., Did the daughter receive something?) were used to assess whether the readers interpreted the sentence literally (i.e., if they chose “no” in this case) or made a noisy-channel inference (if they chose “yes”). These sentences differed in terms of the most likely noise corruption that might be attributed to them (deletion, insertion, exchange). Half of the participants also read a number of sentences containing errors (e.g., A bystander was rescued by the fireman in the *time* of *nick*.), whereas the other half read error-free versions of the sentences (e.g., A bystander was rescued by the fireman in the *nick* of *time*.).We examined the rates of literal interpretation across sentences with distinct edit types (deletion, insertion, exchange) and compared them across populations (IWA vs. control) and exposure conditions (Errors vs. No Errors) using a Bayesian mixture modeling approach.

## Overall analysis approach

Given the nature of the comprehension task, each question has only two possible answers (yes or no), on some proportion of responses, readers might simply guess rather than thoroughly processing the sentence and question. In prior work on healthy participants, accuracy on plausible sentences with unambiguously correct answers was used to exclude participants who were not participating attentively, and the vast majority of participants were above 90% accuracy. However, IWA may be more inclined to guess than the average reader for a number of reasons (e.g., confusion, discomfort, under-confidence) that are not straightforwardly related to their comprehension of the sentences. Using high accuracy on plausible sentences as an inclusion criterion could both discard valuable data and make experimental effects difficult to compare across samples. To examine the rate of literal interpretation and simultaneously account for guessing, we analyze the data from both experiments using a mixture model and estimate its parameters using a Bayesian analysis.

This mixture model assumes that for any given sentence, the person’s response may come from one of two generative processes: 1) A regular reading process in which participants are attempting to extract the intended meaning from the input, or 2) a random guess because they are unable or unwilling to engage in the comprehension task.^3^ The mixing parameter, $z_n$, indicates whether a response comes from the guessing process (when $z_n = 0$) or the regular comprehension process (when $z_n = 1$), as in (2).

Because the outcome is a dichotomous response (Literal or Inference), both processes will be modeled using Bernoulli distributions, which have one parameter (i.e., probability of “success,” which in the case of this comprehension task will be the probability of a literal interpretation). When participants guess on a particular sentence $n$ (i.e., $z_n = 0$), the chance of their response being the (correct) literal interpretation is 0.5 and so is the chance of their response being the (incorrect) noisy-channel interpretation.

2 $$\begin{aligned} Literal_n \sim {\left\{ \begin{array}{ll} Bernoulli (\theta _{Ln}), & \text { if } z_n = 1 \\ Bernoulli (.5), & \text { if } z_n= 0 \end{array}\right. } \end{aligned}$$

When participants are engaged in reading a particular sentence with index $n$, i.e., $z_n = 1$, let the probability of a literal response be $\theta _{Ln}$. In other words, $1-\theta _{Ln}$, would be the probability of a noisy-channel inference. We expect $\theta _{Ln}$ to be affected by multiple aspects of the experimental design as well as individual differences between readers. In order to estimate these effects from the data, we can define a linear model for the value of $\theta _{Ln}$ in log-odds space as in (3).

Given prior work, the model assumes that $\theta _{Ln}$ will differ based on the kinds of edits (deletions, insertions, exchanges) that would need to be posited to recover a plausible sentence from an implausible one. These differences will be captured by $\beta _{ins}$ and $\beta _{exch}$ (with the deletion sentences serving as reference level) and estimated from the data. Larger, more positive values of $\beta _{ins}$ would reflect a higher probability of literal interpretation of a sentence in the insertion condition relative to the deletion condition. Larger, more positive values of $\beta _{exch}$ would reflect a higher probability of literal interpretation of a sentence in the exchange condition relative to the deletion condition. The model also assumes that $\theta _{Ln}$ will differ between populations (healthy control individuals and IWA), as estimated in $\beta _{pop}$. Larger, more positive values of $\beta _{pop}$ would reflect a higher probability of literal interpretation of a sentence among IWA relative to healthy controls. The model also assumes that $\theta _{Ln}$ will be affected by the noise manipulation, as estimated by $\beta _{noise}$ and the interaction of these two, $\beta _{interaction}$. Larger, more positive values of $\beta _{noise}$ would reflect a higher probability of literal interpretation of a sentence in the noise condition. Larger absolute values of $\beta _{interaction}$ would reflect a differential effect of noise across the populations on the probability of literal interpretation of a sentence.

Random variation in the probability of literal interpretation between participants will be captured by $\gamma _{Li}$, allowing for the estimation of a literal interpretation rate fitted to each participant. A larger, more positive value of $\gamma _{Li}$ would reflect a higher probability of literal interpretation of a sentence for participant $i$.

3 $$\begin{aligned} \begin{aligned}&Log(\frac{\theta _{Ln}}{1-\theta _{Ln}}) = \alpha + \gamma _{Li} + \beta _{ins} \cdot insertion_n\\ &\quad + \beta _{exch} \cdot exchange_n \\&\quad + \beta _{pop} \cdot population_n + \beta _{noise} \cdot noise_n\\ &\quad + \beta _{interaction} \cdot population_n \cdot noise_n \end{aligned} \end{aligned}$$

In addition, the mixing parameter $z_n$ itself is defined by the probability of engaging with the task vs. guessing, $\theta _{En}$, as in (4).

4 $$\begin{aligned} z_n \sim Bernoulli (\theta _{En}) \end{aligned}$$

We can also define a linear model for the value of $\theta _{En}$ in log-odds space, see (5). We assume that the probability of engaging, $\theta _{En}$, may be different between IWA and healthy controls. In the current model, we constrain the effect of the population on the rate of guessing, $\beta _{E,population}$, to be negative, to indicate that the rate of guessing is either the same or higher among IWA relative to healthy controls. In addition, there may be random variation in the probability of engaging in the task between participants, which will be captured by $\gamma _{Ei}$. $\gamma _{Ei}$ allows for the estimation of guessing rates for individual participants. A larger, more positive value of $\gamma _{Ei}$ would reflect a higher probability of engaging in the task for participant $i$.

5 $$\begin{aligned} Log(\frac{\theta _{En}}{1-\theta _{En}}) = \alpha _E + \gamma _{Ei} + \beta _{E,population} \cdot population_n \end{aligned}$$

In Experiment 1 and in a follow-up analysis in Experiment 2 with only the IWA, only one population is present so no parameters for effects of the population were estimated. We put mildly informative priors of a normal distribution with a mean of 0 and standard deviation (SD) of 1 on all coefficients ($\beta _{E,population}$ was constrained to be negative on the assumption that IWA are more likely to guess than healthy controls). Variance priors were constrained to be positive by truncating a normal distribution (mean = 0, SD = 0.5 for the participant intercepts). The prior on $\theta _{E}$ was a $Beta(1,1)$ distribution. Full model specifications are available in the OSF repository. As a sensitivity check, all analyses were rerun with wider priors (SD = 2) and the results were unchanged. Posterior distributions for model parameters were obtained using the No-U Turn Sampler in Stan (Stan Development Team, 2022) with four chains of 10000 post-warmup samples (1000 warmup samples discarded). Prior to sampling, adequate parameter recovery was assessed via simulation. Convergence was assessed by examination of the trace plots, which indicated “good mixing,” and the $\hat{R}$ which was close to 1.00. There were 0 divergent transitions. We used the following packages for all data wrangling, visualization and analyses: R (Version 4.4.0; R Core Team, 2021) and the R-packages *cmdstanr* (Version 0.8.1; Gabry & Češnovar, 2022), *papaja* (Version 0.1.2; Aust & Barth, 2020), *posterior* (Version 1.6.0; Vehtari, Gelman, Simpson, Carpenter, & Bürkner, 2021), *tidybayes* (Version 3.0.6; Kay, 2022), and *tidyverse* (Version 2.0.0; Wickham et al., 2019). The stimuli, data, and code to recreate all analyses and figures can be found in the OSF repository: https://osf.io/97c3w/

## Experiment 1

The goal of Experiment 1 was to replicate and extend the results from Gibson et al. (2013) with a different combination of sentences and a novel analysis approach. In particular, the results were expected to replicate the relative rates of literal interpretation across the three edit types (deletion, insertion, exchange) and the reduction in literal interpretation when the overall rate of noise was increased.

### Methods

#### Participants

Forty healthy controls were recruited through Amazon’s Mechanical Turk. They were paid $2.50 for their participation. We restricted the IP addresses to those in the United States. Furthermore, we asked participants what their native language was, and where they were from originally. Payment was not contingent on answers to these questions. All 40 participants self-identified as native speakers of English from the United States. The study was approved by MIT’s Committee on the Use of Humans as Experimental Subjects (COUHES) and performed in accordance with the ethical standards as laid down in the 1964 Declaration of Helsinki and its later amendments.

#### Materials, design, and procedure

Participants read 89 sentences, each of which was followed by a YES/NO comprehension question. 24 of the sentences were test sentences (included in analyses), which were syntactically correct but semantically implausible (e.g., “The mother gave the candle the daughter”). Examples are shown in Table 1. Each participant saw four sentences of each type (double object, transitive, intransitive, prepositional phrase, active, and passive). In addition to the 24 test trials, participants read 30 exposure sentences and 35 filler sentences. Exposure and filler sentences all described plausible events and varied in their sentence structures (e.g., Exposure: “The pirates came to a village in search of gold.” Filler: “A plant is on the window sill.”). The full set of stimuli is available in the OSF repository: https://osf.io/97c3w/

**Table 1:** Example test sentences from each type, with corresponding question, and most likely edit type

Sentence type	Example	Edit
Double Object	The shop sold the bike the student.	Deletion
	Q: Did the student receive something/someone?	
	(Literal: No)	
Transitive	The player benefited the intense practice.	Deletion
	Q: Did the practice benefit from something/someone?	
	(Literal: Yes)	
Intransitive	The sun melted from the snow.	Insertion
	Q: Did the snow melt from something/someone?	
	(Literal: No)	
Prepositional Phrase	The father gave the son to the car.	Insertion
	Q: Did the car receive something/someone?	
	(Literal: Yes)	
Active	The ball kicked the girl.	Exchange
	Q: Did the girl kick something/someone?	
	(Literal: No)	
Passive	The aunt was bought by the book.	Exchange
	Q: Did the book buy something/someone?	
	(Literal: Yes)	

The most plausible *edit* to the sentence was a within-subjects factor. Each participant read eight deletion sentences, eight insertion sentences, and eight exchange sentences.^4^ Two different syntactic structures were used for each edit type.

The *noise* factor was manipulated between subjects (as in Gibson et al., 2013). In the No Noise condition, the 30 exposure sentences were error-free (e.g., A bystander was rescued by the fireman in the nick of time.). In the Noise condition, the 30 exposure sentences contained errors (e.g., A bystander was rescued by the fireman in the *time* of *nick*.).

### Results

Participants responded with high accuracy on filler trials (mean = 92%). Two participants performed at chance but their data were not excluded from analyses in order to keep the analysis approach the same across experiments. We use a mixture model that incorporates an estimate of how likely it is that an individual is guessing.

**Fig. 1:**
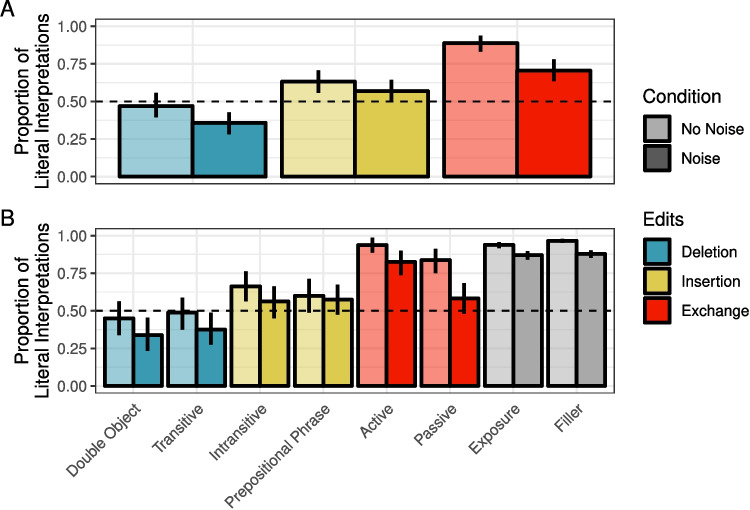
Proportion of correct literal interpretations in Experiment 1 for (**A**) critical trials only across edit and noise conditions and (**B**) all sentence types across noise conditions. *Error bars* reflect bootstrapped 95% confidence intervals over participant means

Readers were least likely to interpret sentences literally when they were the likely result of a word deletion (47%) relative to insertion (63%) or exchange (89%) (see summary in Fig. 1). Across all edit types, the likelihood of literal interpretation was reduced in the Noise condition (Deletion: 36%, Insertion: 57%, Exchange: 70%)

**Fig. 2:**
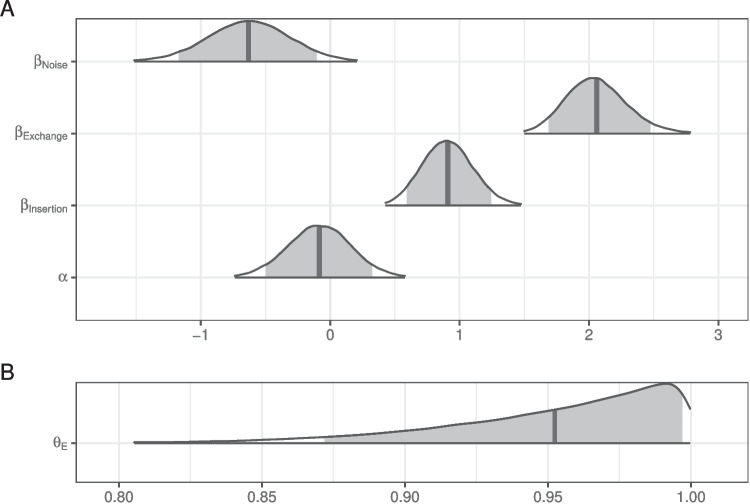
Posterior distributions for fixed effects in the mixture model of literal interpretation in Experiment 1. The density encompasses 99% of the posterior distribution, and the *shaded region* is 90%. The *vertical line* indicates the mean

**Fig. 3:**
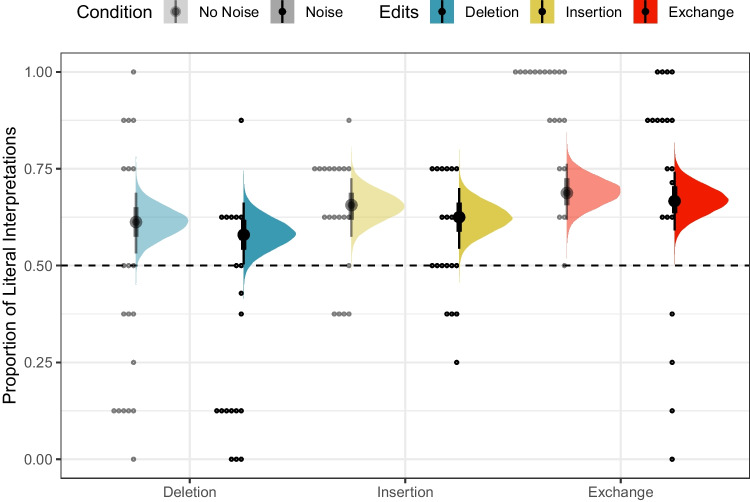
Observed participant means (*dots*) and posterior predictive distributions (*densities*) for condition means from the mixture model of literal interpretation in Experiment 1

The posterior distributions of fixed model parameters are summarized in Fig. 2 and the posterior predictive distributions of condition means are visualized in Fig. 3 alongside the participant means (by condition) in the observed data. Together, they indicate that inference rates are reduced for exchanges and insertions relative to deletions and increased when the overall noise rate increases.

**Fig. 4:**
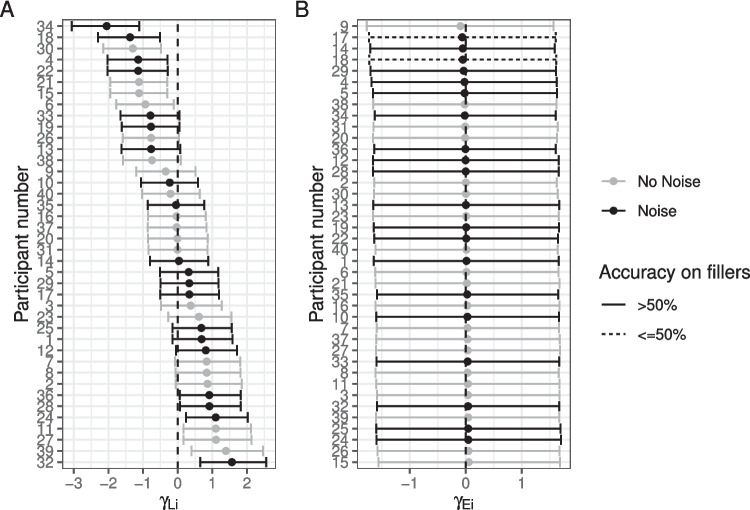
Posterior estimates (mean and 90% credible intervals) for participant-specific tendency to (**A**) interpret test sentences literally and (**B**) engage in the task (as opposed to guess)

The posterior for $\theta _E$ (probability of being engaged in the task) indicates there is very little guessing (Fig. 2B). Figure 4 indicates the posterior estimates for the rate of literal interpretation (A) and guessing rates (B) for each individual. There is substantial individual variability in the rates of literal interpretation (Fig. 4A). Some participants have a tendency to consistently interpret critical sentences literally, while others tend to consistently interpret them non-literally (in line with previous informal observations of individual differences). There is much less individual variability in the tendency to guess (Fig. 4B). Nonetheless, the two individuals who exhibited chance performance on filler trials (dashed lines) are among the four who are estimated to be most prone to guessing, suggesting that both approaches – computing average filler accuracy and estimating a latent tendency to guess with a mixture model – are picking up on the same signal in the data.

### Discussion

The results of Experiment 1 successfully replicate and extend the findings from Gibson et al. (2013) with a new design and analysis approach. Sentences where the most likely edit was a deletion led to more inferences than those where the most likely edit was an insertion, and likely exchanges led to the fewest inferences (most literal interpretations). Adding noise to the environment via exposure trials increased the rate of noisy-channel inferences. Readers were more likely to interpret syntactically correct but semantically implausible sentences according to a non-literal but semantically more plausible meaning when the exposure sentences contained errors. Presumably, the presence of errors in these exposure sentences led the participants to estimate that the probability of noise corruptions was higher. In addition, the model estimated that guessing was rare overall and the two individuals who had performed at chance on filler trials were estimated to have among the highest rates of guessing (on test trials).

## Experiment 2

The goal of Experiment 2 was to investigate noisy-channel inference in IWA and to see whether they adapt to noise in the environment.

### Methods

#### Participants

Twenty-eight individuals with chronic aphasia ( >6 months post-stroke) and 19 (approximately) age-matched controls were recruited to participate at Boston University. They received $10 Amazon gift cards as a thank-you for their time and effort. Because the focus of this study was sentence comprehension and the task required some reading ability, only IWA with relatively moderate aphasia severity were recruited. The primary participation criterion was a score above 55 on the Western Aphasia Battery - Aphasia Quotient (WAB-AQ, Kertesz, 2007). Controls were older on average (mean = 67 years, std. dev. = 11) than IWA (mean = 59 years, std. dev. = 13). See Appendix B for demographic data for individual IWA participants. The study was approved by Boston University’s Institutional Review Board and performed in accordance with the ethical standards as laid down in the 1964 Declaration of Helsinki and its later amendments.

#### Materials, design, and procedure

The materials and design were identical to Experiment 1. In this experiment, participants came into the laboratory at Boston University and completed the study on a laptop while sitting in a quiet room. The trials (sentence and accompanying question) were shown one at a time on the screen. Participants used the “1” and “2” keys to respond “yes” or “no” respectively. The keys were marked with stickers that said “Y” and “N.” During their visit, some participants also completed the Sentence Comprehension subtest (SCT) portion of the Northwestern Assessment of Verbs and Sentences Cho-Reyes and Thompson (2012) (if their NAVS scores were not already collected in the context of a different study within the preceding 6 months). In this task, the participants hear the researcher read a sentence such as “The girl is pulling the boy” and indicate by pointing which of two pictures involving the agents (e.g., The boy pulling the girl in a wheelbarrow vs. the girl pulling the boy in the wheelbarrow) corresponds to the sentence. To visualize how individual differences in performance on the comprehension task relate to performance on the NAVS-SCT, individuals with aphasia were categorized into two groups: “agrammatic” (*N* = 10) and “non-agrammatic” (*N* = 18) based on the NAVS-SCT scores. Participants with accuracy scores below 75% were considered “agrammatic” (Cho-Reyes & Thompson, 2012).

### Results

#### Descriptive statistics for WAB-AQ and NAVS-SCT

Overall aphasia severity (measured by WAB-AQ) and sentence comprehension (measured by NAVS-SCT) were similar across the Noise and No Noise conditions (see Table 2). Individual test scores are available in Appendix B. Any effects of noise condition in the main analysis (below) are unlikely to be attributable to differences between the aphasia severity of participants in each condition.

**Table 2:** Descriptive statistics for IWA across conditions

	NAVS-SCT		WAB-AQ		Age (years)	
Condition	Mean	SD	Mean	SD	Mean	SD
No Noise	0.81	0.15	81.91	11.48	58.17	14.83
Noise	0.80	0.15	81.92	11.71	59.67	12.59

*Note.* 3 IWA missing NAVS-SCT scores

#### All participants

One participant (IWA) only completed 24 of 89 trials. Sixteen participants (all IWA) answered fewer than 24 (out of 35) filler comprehension questions correctly. In other words, their performance was not statistically better than chance (based on a binomial test). In many studies, data from these individuals would be excluded from further analysis. Here we did not exclude any participants’ data and instead use a hierarchical mixture model to simultaneously estimate both comprehension and tendency to guess.

**Fig. 5:**
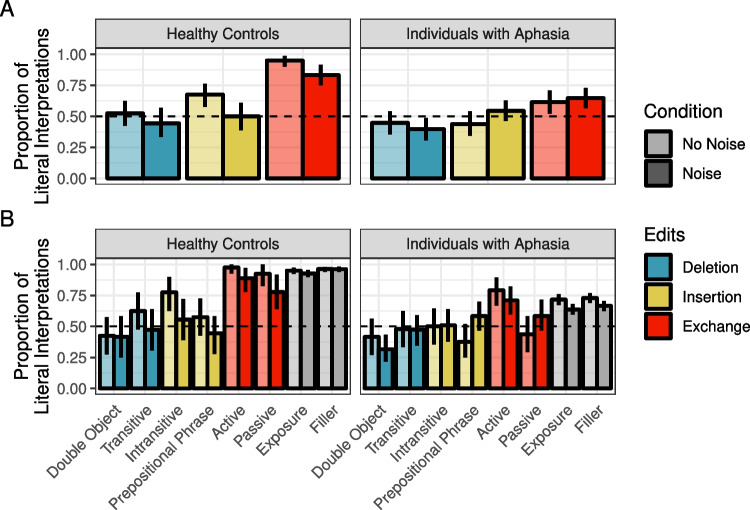
Proportion of correct literal interpretations in Experiment 2 for (**A**) critical trials only across edit and noise conditions and (**B**) all sentence types across noise conditions. *Error bars* reflect bootstrapped 95% confidence intervals over participant means

As in Experiment 1, healthy controls were least likely to interpret sentences literally when they were the likely result of a word deletion (52%) relative to insertion (68%) or exchange (95%) (see summary in Fig. 5). Across all edit types, the likelihood of literal inference was reduced in the Noise condition (Deletion: 44%, Insertion: 50%, Exchange: 83%).

For IWAs, the rates of literal interpretation followed the same pattern across edit types (deletion< insertion< exchange) as those of healthy controls, but they were overall lower. Moreover, the effect of noise appeared to reverse for some edit types, relative to healthy controls. For both insertion and exchange sentences, IWA were numerically more likely to interpret them literally in the Noise condition than the No Noise condition (the opposite pattern was observed for healthy controls). These patterns were driven primarily by prepositional phrase sentences and passive sentences (see Fig. 5B).

**Fig. 6:**
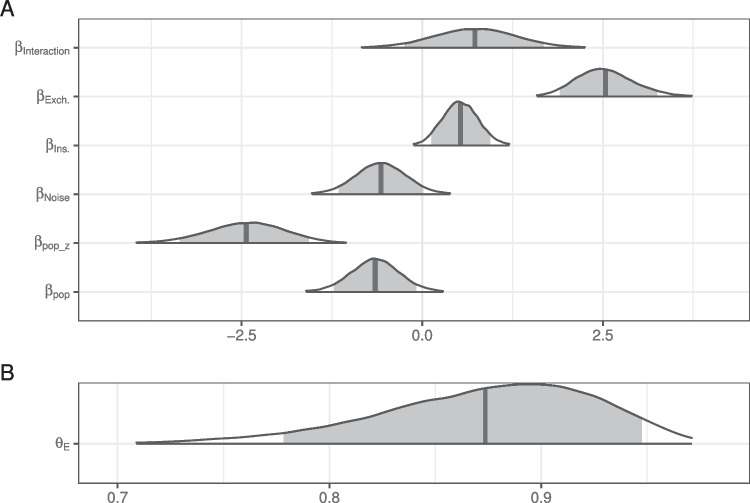
Posterior distributions for fixed effects in the mixture model of literal interpretation in Experiment 2. The density encompasses 99% of the posterior distribution and the *shaded region* is 90%. The *vertical line* indicates the mean

**Fig. 7:**
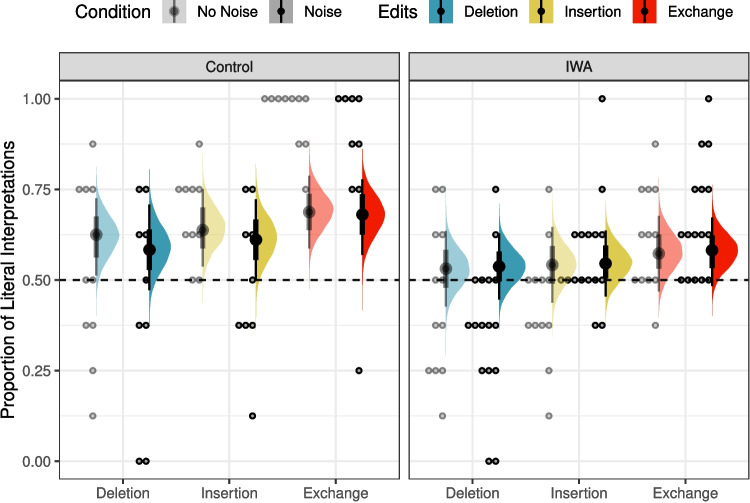
Observed participant means (*dots*) and posterior predictive distributions (*densities*) for condition means from the mixture model of literal interpretation in Experiment 2

The posterior distributions of fixed model parameters are summarized in Fig. 6 and the posterior predictive distributions of condition means are visualized in Fig. 7 alongside the participant means (by condition) in the observed data. Jointly these indicate that the effects of edit type are positive: literal interpretations are more likely for insertions and (even more so) exchanges relative to deletions. The effect of population on the rate of literal inferences is mostly negative so there is moderate evidence for a higher rate of inference among IWA. IWA are more likely to interpret the test sentences according to the non-literal but semantically more plausible meaning than healthy controls. Similarly, the effect of noise condition on the rate of literal inferences (when not guessing) is mostly negative (90% credible interval does not overlap with 0) so there is moderate evidence for a higher rate of inference for healthy controls when they are in the Noise condition. There is little indication of an effect of noise for IWA. IWA appear to make noisy channel inferences at similar rates in both Noise and No Noise conditions. However, the posterior for the interaction is wide and the 90% credible interval contains zero, suggesting that the data are insufficient to address whether the size of the effect of noise differs between controls and IWA.

The posterior for $\theta _E$ (probability of being engaged in the task) indicates there is substantially more guessing than in Experiment 1 (Fig. 6B). The effect of the population on the rate of guessing,$\beta _{pop_z}$, is negative (i.e., patients are more likely to guess) but there is a lot of uncertainty in its estimate.

To further probe the role of the interaction of noise and population, we compare this model to a simpler model, without the interaction term, in terms of their ability to predict held-out data. Comparing these models using leave-one-out cross-validation (Vehtari et al., 2017), we do not see evidence of a difference in the expected log pointwise predictive density ($\Delta$ = -0.38, Std. Error = 0.89 ). The inclusion of the interaction term does not appear to improve the model’s predictive ability; however, for subsequent analyses, we use the model with the interaction term for completeness.

#### Individual differences in noisy-channel inference

**Fig. 8:**
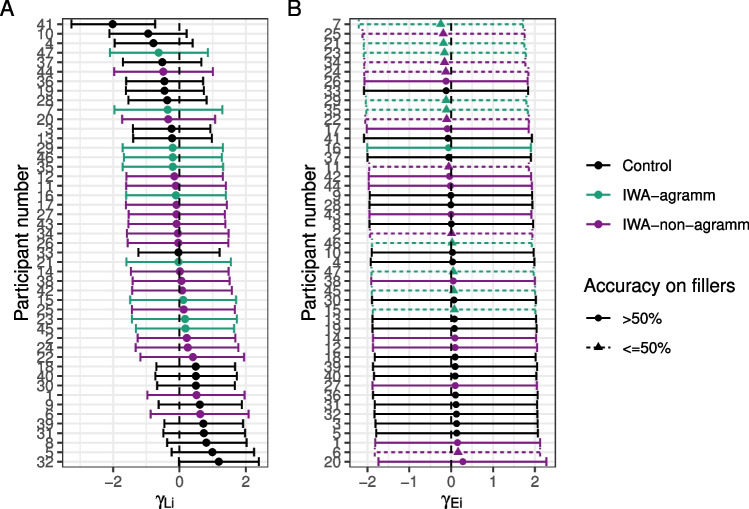
Posterior means and 90% credible intervals for individual adjustments to (**A**) the log-odds of taking the literal interpretation and (**B**) the log-odds of being engaged in the task (rather than guessing)

To further probe individual differences, we plot the posterior estimates of the random intercepts by participant (individual adjustments to the log-odds of taking the literal interpretation). In other words, Figure 8A indicates whether each individual is more or less prone to noisy-channel inference (for deletion sentences).

**Fig. 9:**
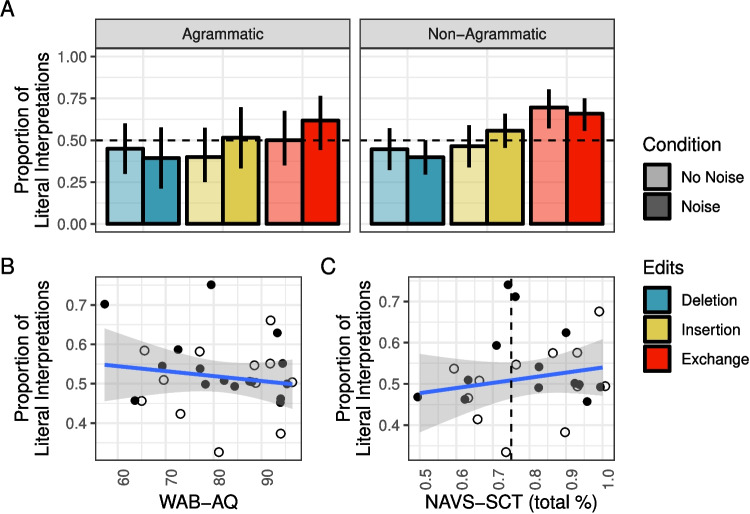
Proportion of correct literal interpretations in Experiment 2 for IWA only (**A**) across edit and noise conditions and (**B**) over WAB-AQ and NAVS-SCT scores (*dashed line* indicates cutoff for “agrammatism”). *Error bars* reflect bootstrapped 95% confidence intervals over participant means. (WAB-AQ = Western Aphasia Battery - Aphasia Quotient; NAVS-SCT = Northwestern Assessment of Verbs and Sentences - Sentence Comprehension Test)

**Fig. 10:**
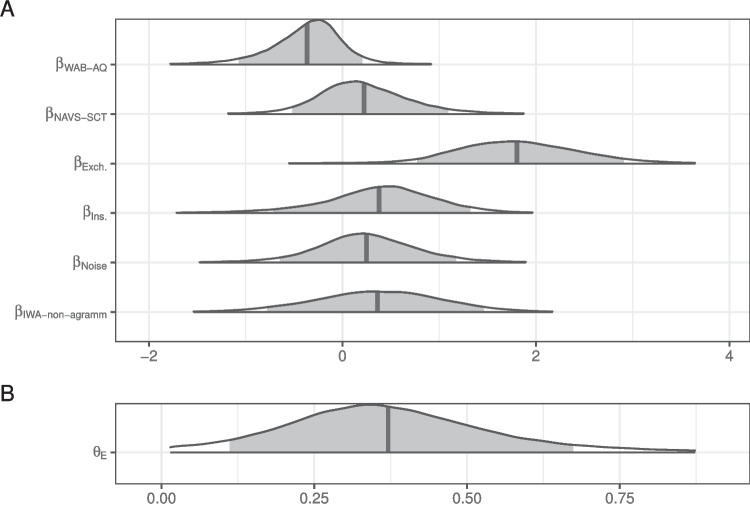
Posterior distributions for fixed effects in the mixture model of literal interpretation for IWA in Experiment 2. The density encompasses 99% of the posterior distribution and the *shaded region* is 90%. The *vertical lines* indicate the means of each distribution. (WAB-AQ = Western Aphasia Battery - Aphasia Quotient; NAVS-SCT = Northwestern Assessment of Verbs and Sentences - Sentence Comprehension Test)

It is interesting to note that many of the more extreme values belong to individuals in the control group, suggesting that they may tend to fall into one of two groups: those who have a consistent tendency to engage in inference and those who consistently treat sentences literally. IWA appear to be less variable and more unimodal on this dimension. It is noteworthy that agrammatic and non-agrammatic IWA are interspersed in their individual tendencies toward literal interpretation, suggesting that this coarse-grained distinction (based on a single score) may not be capturing much meaningful variance in terms of comprehension behaviors in this task. Nonetheless, among those who are most likely to adopt the literal interpretation, there are few IWA classified as agrammatic (among the 15 participants with the most positive tendencies toward literal interpretation, five are non-agrammatic IWA and one is an agrammatic IWA), consistent with previous reports that they are more likely to rely on semantic plausibility.

Further, we can look at how likely each individual is to guess relative to the overall intercept (Fig. 8B). Dashed lines and triangles indicate participants who had chance accuracy on fillers (by binomial test). Almost all of those subjects have the most negative individual intercept effects indicating that the model is able to estimate individuals’ tendency to guess. Interestingly, participant number 6 performed near chance on filler trials and their data would have been discarded with a standard approach, yet the current model estimates that their engagement during critical sentences was higher than many controls’. Among those who are most likely to be engaged (and not guessing), there are no IWA classified as agrammatic. Agrammatic IWA may find the task more challenging than non-agrammatic IWA.

#### IWA only

In order to further probe any possible relationships between aphasia, syntactic processing, and non-literal sentence interpretation, we conducted a follow-up analysis including only IWA for whom both WAB-AQ and NAVS-SCT scores were available (*N* = 25). Figure 9 shows the rates of literal interpretation by condition for each subgroup of IWA (Fig. 9A) as well as their relationship to aphasia severity (Fig. 9B) and agrammatism (Fig. 9C). The effect of edit type on literal interpretation rates appears to hold in both agrammatic and non-agrammatic IWA, and agrammatic IWA appear to have somewhat lower rates of literal interpretation than non-agrammatic IWA (Fig. 9A).

Aphasia severity (as measured by WAB-AQ) did not appear to be correlated with an increased rate of noisy-channel inference (Fig. 9B). Similarly, syntactic comprehension performance (as measured by NAVS-SCT) did not appear to be correlated with an increased rate of noisy-channel inference (Fig. 9C). Standardized WAB-AQ and NAVS-SCT scores and agrammatism classification were included as additional predictors of literal interpretation rate in the same model as was used above (see Stan files on OSF for details).

The posterior distributions of fixed model parameters are summarized in Fig. 10. As with the healthy controls in Experiments 1 and 2, sentences with likely exchanges elicit more literal interpretations than those with deletions. In contrast, insertions did not increase the rate of literal interpretation in this population and noise did not appear to decrease it. There was also very little evidence for non-zero effects of agrammatism classification, NAVS-SCT, or WAB-AQ scores. A similar result is observed for a model in which agrammatism classification is not included but is otherwise identical. Better performance on the NAVS-SCT is numerically associated with a higher likelihood of literal interpretation but there is substantial uncertainty around this effect ($\beta _{\text {NAVS-SCT}}$ = $0.35$, 90% CrI = [$-0.24$, $1.07$]).

### Discussion

In two experiments, we have successfully replicated findings from Gibson et al. (2013) indicating that readers often interpret implausible but syntactically correct sentences according to a more plausible non-literal meaning. In particular, and consistent with predictions of the noisy-channel framework, readers are more likely to do so in situations when a corruption responsible for the perceived implausible sentence is likely, either due to the properties of the sentence and its closest alternatives (i.e., whether it requires positing a deletion, insertion, or exchange) or to an increase of the probability of errors in the environment. The use of a Bayesian hierarchical mixture model reveals, among other things, important individual differences in readers’ tendencies to adopt literal vs. non-literal interpretations.

In the second experiment, we also replicated the findings that individuals with aphasia make inferences at a higher rate and they are more likely to do so when the corruption is a more likely one in terms of Levenshtein distance between the perceived sentence and its alternative (Gibson et al., 2016; Warren et al., 2017). However, the manipulation of noise in the environment did not have a straightforward impact on rates of inference for IWA. Healthy controls appeared more likely to make inferences when the likelihood of noise was increased in both Experiment 1 and Experiment 2. This effect was less apparent in IWA, but the uncertainty in the estimate of the interaction term (which could reflect limitations of either the data or the model or both) does not allow for a strong conclusion regarding whether the effect of noise is in fact smaller for IWA than healthy controls. Aphasia severity (as measured by WAB-AQ) and agrammatism classification (as well as the continuous NAVS-SCT score used to classify individuals as agrammatic) did not appear to robustly predict the likelihood of engaging in noisy-channel inference.

## General discussion

Our data and analysis approach extended the prior work in multiple ways. First, we recruited a larger sample of IWA (*N* = 28) than the preceding studies (*N* = 8 in Gibson et al. 2016 and *N* = 16 in Warren et al. 2017). Second, we used a reading task rather than auditory presentation, demonstrating that noisy-channel processing in IWA is not modality-specific. Third, and most importantly, we use a latent mixture modeling approach to account for the fact that rates of guessing are likely to differ between healthy controls and IWA. The analysis provides insights into individual differences, in both healthy readers and IWA, in terms of sentence interpretation and guessing tendencies. Understanding the source of these individual differences in interpretation is an interesting avenue for future research.

The modeling provides converging evidence that IWA are more likely to make noisy-channel inferences than controls, even when differential rates of guessing are accounted for. An additional advantage of this analysis approach is that we avoid excluding a large number of participants based on an arbitrary criterion (accuracy threshold on filler trials). In a follow-up analysis, we observed that aphasia severity and syntactic comprehension ability, as measured by WAB-AQ and NAVS-SCT did not appear to predict the rate of inferences, consistent with prior work reporting no significant correlations between inference rates and measures of semantic or syntactic ability (Warren et al., 2017). This is not altogether surprising since we explicitly restricted the range of possible WAB-AQ scores (to >55) and the sample size (*N* = 25 IWA with both scores) is small for detecting a correlation, despite being larger than previous work.

Contra Warren et al. (2017), we do not interpret the lack of a correlation as being inconsistent with a noisy-channel account of aphasic language comprehension. They argued that the noisy-channel framework predicts a linear relationship between amount of syntactic impairment and rate of non-literal inference at the level of the individual. This is not necessarily the case. In our view, the probability of making a noisy-channel inference is guided by stable individual differences (e.g., IWA are generally more likely to make noisy-channel inferences than healthy adults but there is substantial variability within both populations), stimulus properties (e.g., the prior probabilities of the sentence at hand and any close alternatives, the probabilities of any potential noise corruptions), and broader contextual factors (e.g., the probabilities of noise corruptions in a given environment). How the neurological condition underlying aphasia is related to any of these components is a complex and open question.

One possibility is that each individual’s estimate of the probability of noise tracks the amount of noise that they are exposed to in everyday life, which could be affected by a number of things (how many other individuals with aphasia they interact with, what production or perceptual errors they themselves make, etc.). Previous work shows that healthy adults adapt their noise estimate to the environment (Gibson et al., 2013; Ryskin et al., 2018). Though we did not observe evidence of adaptation among IWA in the present experiment, this learning may operate over a longer timescale. This suggests that there may be variability across individuals in terms of the relative probabilities they assign to different kinds of noise corruptions and this could impact which sentences they are more likely to interpret non-literally. The measures used to assess syntactic impairment (e.g., accuracy on the NAVS) treat all incorrect interpretations as equivalent (one point is subtracted from the participant’s score) without accounting for differences in the probability of noise corruptions across different structures (e.g., some structures require positing a deletion whereas others may require positing multiple insertions and deletions in order to arrive at a more plausible alternative). These assessments also rely on pairs of interpretations, which do not differ in their semantic plausibility (e.g., A girl pulling a boy vs. a boy pulling a girl) so the prior probability of each sentence reflects the frequency of the structure (e.g., a subject-extracted relative clause may have higher prior probability than an object-extracted relative clause) rather than semantic plausibility. In the current work we have assumed that priors related to event plausibility are not impacted by aphasia whereas the noise likelihood could be. On the other hand, it is an open question whether it can be assumed that structural priors are unaltered in aphasia. A more detailed analysis of the patterns of interpretations across sentence types/structures may reveal individual differences in the kinds of errors that readers (with and without aphasia) make and consequently the kinds of noise and structures they consider probable. This could clarify the relationship between syntactic impairment diagnostic scores and noisy-channel inferences, however, it would likely require a much larger dataset (both in terms of participants as well as items) and is beyond the scope of the current work.

An alternative possibility is that the relationship between noise and syntactic impairment is thresholded: any individual with aphasia expects more noise in their input because they are aware that they have received a diagnosis of aphasia and that amount of increase is similar across people regardless of severity. The observation that there appeared to be less variability in the individual tendencies to make inferences among IWA than healthy controls (Fig. 8A) may be consistent with this speculation. Future efforts to measure whether IWA adapt to the noise in their input with greater precision may help to constrain this hypothesis space.

### Noisy-channel and alternative accounts for “agrammatic” comprehension

The current data are largely inconsistent with an account on which agrammatic comprehension in aphasia reflects damage to a syntactic module (e.g., Grodzinsky, 1990). Healthy adults, with presumably intact syntactic knowledge, vary substantially in their tendency to rely on the literal, syntax-driven interpretation or the semantically plausible interpretation (Fig. 4A). The response patterns of IWA across sentence types are qualitatively similar those of healthy readers (Fig. 5) and some IWA are more likely to follow the syntax-driven interpretation than some healthy controls (Fig. 8A). These results are consistent with prior work, briefly reviewed in the introduction, indicating that important aspects of syntactic knowledge are preserved even in IWA whose behavior appears “agrammatic” (e.g., Saffran & Schwartz, 1994). The noisy-channel perspective on aphasic comprehension echoes similar proposals regarding the role of rational, adaptive behaviors in aphasic production (e.g., dropping low-information-content function words in speech) giving rise to the appearance of a deficit in syntactic abilities (Fedorenko et al., 2023).

In contrast, the current data do not speak directly to the role that either working memory or cognitive control might play in healthy or agrammatic sentence comprehension. Working memory demands were intentionally minimized in the studies by making the sentences available for as long as readers wanted. In principle, a role for cognitive limitations in sentence processing is entirely compatible with the noisy-channel framework. For instance, noisy memory for the prior context in a sentence explains patterns of errors in production and online processing difficulties in healthy readers (Hahn et al., 2022). To our knowledge, no specific proposal for the role of cognitive control in noisy-channel inference has been put forth yet, but these ideas are also, in principle, compatible. We can speculate that cognitive control may be involved in the process of considering multiple alternative intended sentences or inputs and their relative posterior probabilities. Previous electrophysiologic evidence indicates that the P600 – an event-related potential which has been tied to cognitive control (Ovans et al., 2022) – appears greater when readers are more likely to make a noisy-channel inference and interpret linguistic input according to a close plausible alternative (Ryskin et al., 2021). This sketch of a proposal for the role of cognitive control in noisy-channel inference shares commonalities with previous proposals that cognitive control resolves competition between semantic and syntactic interpretations of the stimulus (e.g., Thothathiri et al., 2023), though in the case of noisy-channel there are many more alternatives, each reflecting a different possible noise corruption of an intended sentence. We leave it to future work to elaborate on these proposals and investigate whether they make distinct and testable predictions.

## Conclusion

In sum, the current work lends further support to a construal of aphasic language comprehension as obeying the same overall principles of the noisy-channel framework that have been reported in a large number of studies of healthy adult language processing (Futrell et al., 2020; Gibson et al., 2013; Gibson et al., 2017; Keshev & Meltzer-Asscher, 2021; Levy, 2008, 2011; Levy et al., 2009; Norris & Kinoshita, 2012; Poliak et al., 2023; Poppels & Levy, 2016; Ryskin et al., 2018, 2021; Zhang, Ryskin, & Gibson, 2023). A key difference is that the noise model in aphasia appears to assign a greater likelihood to any kind of noise in the input. The relative likelihoods of different noise operations (deletions>insertions>exchanges) appear to be preserved, but it is unknown whether more fine-grained differences exist between and within populations. Further, it remains unclear to what extent the noise model is malleable in IWA the way that it has been shown to be in healthy adults (Gibson et al., 2013; Ryskin et al., 2018). Pursuing this noisy-channel explanation has the potential to yield insights about the cognitive processes involved in everyday language comprehension in health and disease as well as have implications for clinicians who may target their efforts to supporting their patients’ ability to make rational inferences that are adapted to their environment.

## Figures and Tables

**Fig. 11:**
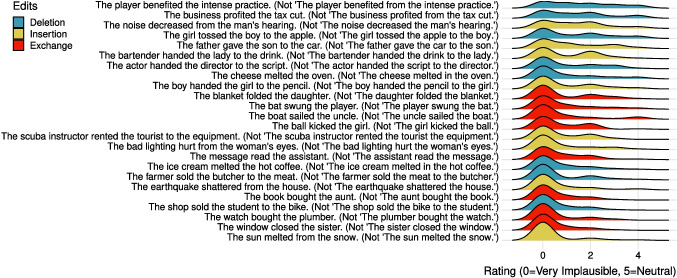
Densities of implausibility ratings by prompt. *Color* indicates the edit type for each critical sentence (as the sentence appears in the main experiments)

**Fig. 12:**
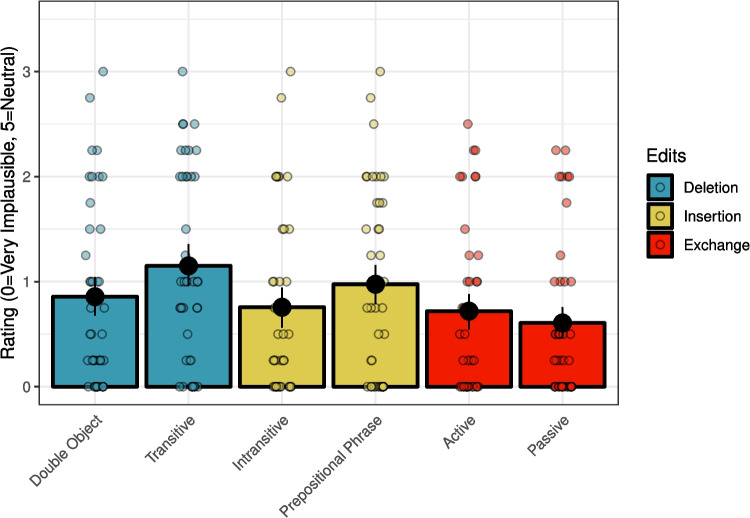
Implausibility ratings by sentence and edit type. *Colored points* indicate participant means. *Error bars* around the mean indicate bootstrapped 95% confidence intervals over participants means

## Data Availability

The data, materials, and code to recreate all analyses and figures are available at the following link: https://osf.io/97c3w/.

## Methods

### Participants

Forty participants were recruited on the Prolific platform. All participants were located in the US or UK, reported using English as their primary language, and had no language-related disorders. The study lasted approximately 5 min and each participant was paid $1.00.

### Procedure & materials

Participants were asked to use a Likert scale (0 = Very Implausible, 3 = Implausible, and 5 = Neutral) to rate how implausible the events described in 24 sentences were. Participants were also told that the sentences might be similar to sentences describing more plausible events, but they should rate the plausibility of the given sentence, not the alternative. The alternative was presented alongside each implausible sentence for clarity (e.g., “The shop sold the student to the bike. (Not ‘The shop sold the bike to the student.’)). Sentences with non-canonical sentence structures were presented in the canonical form to avoid creating any additional comprehension difficulty for participants unrelated to their judgments of the semantic plausibility of the events. For instance, passive sentences were presented in active form. The complete set of prompts and the distribution of ratings is summarized in Fig. 11.

## Results

Implausibility ratings by sentence type and edit type are summarized in Fig. 12. Rating responses were modeled in a Bayesian multilevel linear model using the brms (Bürkner, 2017) frontend for Stan. Edit type was included as a fixed predictor. Varying intercepts were included for participants along with varying slopes for edit type by participant. Default priors were used for all parameters (flat priors for $\beta$ coefficients and Student’s t(df = 3, $\mu$ = 0, $\sigma$=2.5) for the intercept and all variance parameters). Estimated Marginal Means (*EMM*s) and pairwise comparisons ($\Delta$) were extracted using the emmeans package (Lenth, 2024).

Deletion (*EMM*= 0.99) sentences and Insertion sentences (*EMM*= 0.86) were rated as similarly implausible ($\Delta$ = 0.14, *HPDI* = [-0.05, 0.32]). Exchange sentences (*EMM*= 0.66) were rated as more implausible than deletion sentences ($\Delta$ = 0.34, *HPDI* = [0.19, 0.49]). Exchange sentences were also rated as somewhat more implausible than insertion sentences ($\Delta$ = 0.20, *HPDI* = [0.03, 0.39]).

## Appendix B

**Table 3:** Individual participant scores and ages

Participant	Condition	NAVS-	WAB-	Age	Sex	Group
ID		SCT	AQ			
1	No Noise	1.00	91.90	59.00	M	nAG
2	No Noise	0.93	77.10	43.00	F	nAG
6	Noise	0.77	57.30	24.00	F	nAG
7	No Noise	0.67	73.10	26.00	M	AG
11	Noise	NA	84.40	64.00	M	MISSING
12	Noise	0.93	87.50	48.00	Other	nAG
14	Noise	0.83	77.20	51.00	M	nAG
15	Noise	0.73	79.50	72.00	M	AG
16	Noise	0.63	78.30	63.00	M	AG
17	No Noise	0.93	96.40	54.00	F	nAG
20	Noise	0.97	93.90	56.00	F	nAG
21	No Noise	0.67	69.60	65.00	M	AG
22	Noise	0.90	93.30	NA	M	nAG
23	No Noise	0.60	88.50	73.00	F	AG
24	No Noise	0.87	65.60	70.00	F	nAG
25	Noise	NA	69.30	64.00	M	MISSING
26	Noise	1.00	88.00	73.00	F	nAG
27	No Noise	1.00	88.80	71.00	F	nAG
29.00	Noise	0.63	63.60	74.00	F	AG
34.00	Noise	0.93	82.20	60.00	M	nAG
35.00	Noise	0.50	94.00	66.00	M	AG
38.00	No Noise	0.77	91.80	48.00	M	nAG
42.00	Noise	NA	94.40	65.00	M	MISSING
43.00	Noise	0.83	95.30	52.00	M	nAG
44.00	No Noise	0.90	94.00	53.00	M	nAG
45.00	Noise	0.70	72.60	63.00	M	AG
46.00	No Noise	0.63	65.00	57.00	M	AG
47.00	No Noise	0.73	81.10	79.00	M	AG

(WAB-AQ = Western Aphasia Battery - Aphasia Quotient, NAVS-SCT = Northwestern Assessment of Verbs and Sentences - Sentence Comprehension Subtest, Age in years, AG = Agrammatic, nAG = Non-agrammatic IWA)

## Footnotes

1 In this example and throughout the current paper, we focus on the role of semantic plausibility in the prior probability of a sentence. In particular, sentences that are implausible given world knowledge (e.g., involving an animate candle) are assumed to have lower prior probability relative to sentences that are semantically plausible. However, in the noisy-channel framework, the prior probability of a sentence is determined by other factors as well. For instance, a sentence with a rare syntactic structure will generally have lower prior probability than a sentence with a more common structure and readers may be more likely to infer that an alternative (more syntactically probable) sentence was intended when they read a sentence with a rare syntactic structure (Poliak, Ryskin, Braginsky, & Gibson, 2023). The preceding context can affect the prior probability of a sentence as well (Chen, Nathaniel, Ryskin, & Gibson, 2023). For instance, in a supportive discourse context, even a sentence describing an anthropomorphic candle can become highly probable (see Nieuwland & Van Berkum, 2006).

2 The Levenshtein distance (Levenshtein, 1966) is a metric that quantifies how (dis)similar two character sequences are. It corresponds to the minimum number of single-character edits (insertions, deletions, or substitutions) required to change one sequence into the other. The Levenshtein distance is 0 when the sequences are identical. In the noisy-channel framework, $P(S_p|S_i)$ is typically greater when $S_i$ and $S_p$ have a smaller Levenshtein distance, capturing the idea that a small number of corruptions is more likely than many corruptions happening at once to one message.

3 This is a simplifying assumption. In reality, it is likely that many guesses start out as genuine attempts to understand the sentence that are then abandoned.

4 The test sentences were normed for semantic implausibility with an independent set of participants, who did not take part in Experiment 1 or 2. The sentences from the three edit types were not perfectly matched in their semantic implausibility. Participants rated the exchange sentences (e.g., “The ball kicked the girl”) as more implausible relative to deletion sentences and (marginally) insertion sentences (e.g., “The father gave the son to the car”). This suggests that the materials constitute a very conservative test of the hypothesis that readers will make more inferences when the probability of noise is higher, because the greater semantic implausibility of the exchange sentences should make them *more* likely to be interpreted non-literally, whereas the lower probability of the exchange errors/edits should make them *less* likely to be interpreted non-literally. Full details and results of the norming study are reported in Appendix A.

## Code availability

The data, materials, and code to recreate all analyses and figures are available at the following link: https://osf.io/97c3w/.

## Declarations

### Open Practices Statement

The data, materials, and code to recreate all analyses and figures are available at the following link: https://osf.io/97c3w/?view_only=66e72ab3363b4229b862fc92212cb5bd The experiments were not preregistered.
